# An Efficient Index for Reachability Queries in Public Transport Networks

**DOI:** 10.1007/978-3-030-54832-2_5

**Published:** 2020-07-08

**Authors:** Bezaye Tesfaye, Nikolaus Augsten, Mateusz Pawlik, Michael H. Böhlen, Christian S. Jensen

**Affiliations:** 8 ERIC (Research Unit 3083), Université Lumière Lyon 2, Lyon, France; 9 National Research University, Higher School of Economics, St. Petersburg, Russia; 10 Poznan University of Technology, Poznań, Poland; 11 University of Salzburg, Salzburg, Austria; 12 University of Zurich, Zürich, Switzerland; 13 Aalborg University, Aalborg, Denmark

**Keywords:** Reachability queries, Public transport networks, Temporal graphs, Spatial network databases

## Abstract

Computing path queries such as the shortest path in public transport networks is challenging because the path costs between nodes change over time. A reachability query from a node at a given start time on such a network retrieves all points of interest (POIs) that are reachable within a given cost budget. Reachability queries are essential building blocks in many applications, for example, group recommendations, ranking spatial queries, or geomarketing. We propose an efficient solution for reachability queries in public transport networks. Currently, there are two options to solve reachability queries. (1) Execute a modified version of Dijkstra’s algorithm that supports time-dependent edge traversal costs; this solution is slow since it must expand edge by edge and does not use an index. (2) Issue a separate path query for each single POI, i.e., a single reachability query requires answering many path queries. None of these solutions scales to large networks with many POIs. We propose a novel and lightweight reachability index. The key idea is to partition the network into cells. Then, in contrast to other approaches, we expand the network cell by cell. Empirical evaluations on synthetic and real-world networks confirm the efficiency and the effectiveness of our index-based reachability query solution.

## Introduction

We study the problem of scalable and efficient reachability querying in public transport networks. A reachability query retrieves all points of interest (POIs) reachable from a given query node at a specific start time within a given time budget. The start time is required since the reachability result changes over time. Interesting applications of reachability queries include group recommendations, ranking spatial queries, urban planning, and geomarketing. We present two examples.

Consider a platform that recommends events to a group of people such that the group members like to attend the event together [2, 14]. Group members are query nodes and events are POIs. When the group is given, the events must be evaluated by various criteria to optimize the benefit to the group. One important aspect is the location of the event relative to the group members. The start time and the travel time budget to reach an event may differ for each member. Events too far away are unlikely to be successful. A single recommendation comprises multiple reachability queries, one for each group member.

Another example is a real estate website that ranks properties (query nodes) according to user preferences. The users may customize reachability criteria for different POIs (e.g., school, working place, train station). Thereby, the time budget for individual types of POIs may vary: a user may be willing to commute to work for an hour, while a school must be nearby. Ranking the results of a single user query requires the computation of multiple reachability queries: one for each property and parameter setting.

To support such applications, reachability queries must be computed efficiently. Achieving this goal in public transport networks is tricky since the shortest path between two nodes depends on the start time, and the time to traverse a path may vary greatly across time. In a public transport network, stations are nodes, and connections between stations are edges between nodes. An edge can only be traversed at specific points in time as given by a schedule. Therefore, computing an index for public transport networks is more complex than for networks with constant edge-traversal costs or networks in which an edge can be traversed at any time (like pedestrian networks or road networks).

### Example 1

**Fig. 1.**
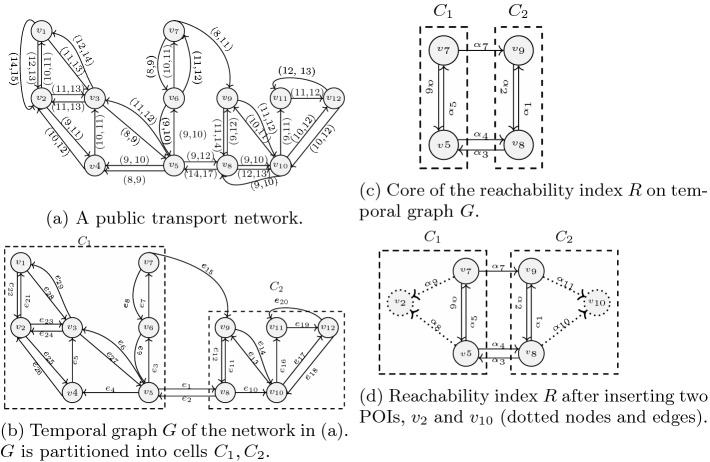
Temporal graph of public transport network and reachability index.

Consider, the public transport network in Fig. 1a. The nodes $v_{1}, v_{2},$ $\ldots , v_{12}$ represent stations, and the directed edges represent connections between the stations. Each connection has a pair $(t_d, t_a)$ of departure and arrival times. For example, there is a connection leaving $v_4$ at time 10 and arriving at $v_3$ at time 11. The traversal cost between nodes is expressed in terms of time units. The cost of traversing the edge $(v_4, v_3)$ at time 9 is 2, since we have a waiting time in addition to the edge traversal time. The shortest path from $v_{10}$ to $v_{11}$ at start time $t_{s}=9$ has cost 2 (edge $(v_{10},v_{11}$)), while at $t_{s}=10$, the cost of the shortest path is 3 (edges $(v_{10},v_{12})$, $(v_{12}, v_{11})$). At start time $t_{s}=9$, the nodes $\lbrace v_{8},v_{9},v_{11} \rbrace$ are reachable from $v_{10}$ with budget $\varDelta t=2$; at $t_{s}=10$ with the same budget, we can reach the nodes $\lbrace v_{9},v_{12} \rbrace$.

The state of the art in answering reachability queries in public transport networks includes two approaches. The first is based on a temporal version of Dijkstra’s algorithm [10] that expands in the network until the budget is exhausted. Algorithms following this approach compute a so-called isochrone (the reachable region) and intersect it with the set of POIs [6, 12]. Since all edges in the isochrone must be expanded, these algorithms do not scale to large networks. The second approach translates a single reachability query into a set of path queries (e.g., shortest path or earliest-arrival path [18, 20, 21]), one for each POI. Path queries require heavy index structures and do not scale to large numbers of POIs.

We propose an index-based technique for reachability queries in public transport networks. Instead of expanding edge by edge, in a precomputation step, we partition the network into cells and construct a novel reachability index. At query time, the index is used to expand cell by cell. Each cell covers a region of the network and all POIs in that region. The precomputation effort for a specific cell is independent of the other cells such that the index scales to large networks. The index is small, even smaller than the original graph for some inputs. To the best of our knowledge, this is the first work that proposes an index for reachability queries in public transport networks.

The rest of the paper is structured as follows. In Sect. 2, we define the problem, and we give an overview of our solution in Sect. 3. We introduce our reachability index in Sect. 4 and discuss query processing using the index in Sect. 5. In Sect. 6, we review related work. In Sect. 7 we investigate experimentally the performance of our solution. We conclude in Sect. 8.

## Preliminaries and Problem Definition

In a public transport network, stations are nodes and connections are edges. A connection has a departure time $t_d$ and an arrival time $t_a$. We assume periodic schedules as is typically the case in public transport networks, e.g., schedules repeat daily or weekly.

A *temporal graph* $G=(V,E,c)$ is a directed graph with vertices *V*, edges $E\subseteq V\times V$, and a time-dependent cost function *c*(*e*, *t*), $c:E\times \mathbb {R}\rightarrow \mathbb {R}_{\ge 0}$ that captures the cost of traversing edge *e* starting at time *t*. We represent public transport networks as temporal graphs with a specific cost function, which we derive from the schedule. Each station is a node in the graph, and there is an edge from node *u* to node *v* iff there is a direct connection (i.e., there are no intermediate stops) from the station of *u* to the station of *v*. The cost function is periodic with period $\varPi$, i.e., $c(e,t)=c(e,t+\varPi )$ and piecewise linear; all linear pieces have slope $k=-1$; the cost function is not continuous; all discontinuities are at departure times of some connections. For a single connection $s_i=(t_d,t_a)$ on an edge *e*, the cost in the period $(t_d-\varPi ,t_d]$ is $c_i(e, t)=t_a-t$; if there are multiple connections $S=\{s_1, s_2, \ldots , s_i\}$ for edge *e*, the cost of *e* at time *t* is the minimum of all costs of the individual connections at time *t*, $c(e, t)=\min \{c_i(e, t)\mid c_i \text { is the cost function of connection } s_i\}.$ Our cost function is *consistent*, i.e., for any edge $e\in E$ and all start times $t_1\le t_2$: $t_1+c(e, t_1)\le t_2+c(e, t_2)$. Intuitively, in a consistent cost function, it never pays off to wait. Consistency is required for the use with Dijkstra’s shortest-path algorithm [17].

### Example 2

Consider the edge $e_{7}$ in Fig. 1b with connections $s_1=(10,11)$ and $s_2=(11,12)$. Then, for $\varPi =12$, the cost function of $s_1$ is $c_1(e_{7},t)=11-t$, $t \in (-2, 10]$ and for $s_2$ is $c_2(e_{7},t)=12-t$, $t \in (-1, 11]$. The overall cost function $c(e_{7},t)$ is the minimum of $c_{1}(e_{7},t)$ and $c_{2}(e_{7},t)$.

A *path* *p* from *u* to *v* in a temporal graph $G=(V, E, c)$ is a sequence of edges $p=\langle e_1, e_2, \ldots , e_n\rangle$ such that $e_i \in E$, $e_i=(w_{i-1}, w_i)$, $w_0=u$, and $w_n=v$; *P*(*u*, *v*) is the *set of all paths* from node *u* to node *v*. The cost of a path is the fastest time to traverse the path at a given start time. Due to the consistency property of our cost function, the path cost is the sum of all edge costs. The *cost of path* $p=\langle e_1,e_2,\dots ,e_n \rangle$ at time *t*, is the cost sum of all edges in *p*: $c(p,t) = \sum _{1\le i\le n}c(e_i,t_i)$, where $t_1=t$ and $t_i=t_{i-1}+c(e_{i-1},t_{i-1})$ for $i>1$. The *shortest-path cost* from node *u* to node *v* at time *t* is the minimum cost of any path from *u* to *v*, $sp(u,v,t)=\min \{c(p,t) \mid p\in P(u,v) \}$. A path with the minimum cost is called the *shortest path*. A node *v* is *reachable* from a node *u* at time *t* within budget $\varDelta t$ iff there is a path $p\in P(u,v)$ such that the cost of *p* at time *t* is no larger than $\varDelta t$, i.e., $c(p, t)\le \varDelta t.$ The *reachability query*, $RQ(u,t,\varDelta t) = \{ v\in V \mid \exists p\in P(u,v), c(p,t)\le \varDelta t, v \in POI \}$, in a temporal graph $G=(V, E, c)$ with points of interst $POI \subseteq V$, returns all points of interest reachable from node *u* at time *t* within budget $\varDelta t$.

*Problem Definition.* The goal of this work is to develop an efficient index-based solution for reachability queries that scales to large temporal graphs.

## Solution Overview

We propose a novel index structure, the *reachability index*, to answer reachability queries. We introduce a bulk loading technique for our index, provide access methods for answering reachability queries, and discuss the incremental insertion and deletion of POIs in the index.

The reachability index is built in a precomputation step. To construct the index, we partition the temporal graph into disjoint cells. Any such partitioning yields correct results. The choice of cells, however, affects the effectiveness of the index. We define requirements for a good partitioning and propose a suitable partitioning technique.

The index is a temporal graph that contains only those nodes of the original graph that are POIs or directly connect different cells, called *border nodes*. Each POI belongs to a cell. POIs can be inserted into and deleted from the index at any time; the update cost is low and depends on a single cell. The index consists of the original edges between border nodes of neighboring cells and new edges between the border nodes within a cell. Further, an edge between each POI and the border nodes in its cell is introduced. The edge costs are the costs of shortest paths between the respective nodes in the original graph.

A high number of border nodes per cell increases the index size. Each POI adds as many edges to the index as there are border nodes in its cell.

A search query traverses the index cell by cell. The border nodes are used to cross cells and to reach neighboring cells. For each border node, we verify if any of the POIs in that cell is reachable.

## The Reachability Index

The reachability index *R* is a temporal graph that is constructed from the original graph *G* as follows:

1. *Graph partitioning.* The nodes of graph *G* are split into disjoint *cells*. At query time, instead of expanding edge by edge in *G*, we expand cell by cell in the index.
2. *Constructing the index core.* Based on the graph partitioning, we insert nodes and edges into the initially empty index. This index core never changes.
3. *Computing the index cost function.* The edge cost is computed as a shortest-path cost for each departure time from a source node to a destination node.
4. *Inserting POIs.* Inserting a POI into a cell adds a new node and an edge to each border node of the cell. POIs that are not *border nodes* can be inserted and deleted dynamically without modifying the rest of the index.

We detail each step of the index construction next. Additionally, we discuss the factors that affect the size of the reachability index and present a compaction technique to reduce the number of connections.

### Graph Partitioning

We partition the nodes of a temporal graph $G=(V,E,c)$ into a set of disjoint cells $C=\{C_1,C_2,\dots ,C_n\}$, such that each node of *G* belongs to exactly one cell $C_i$, i.e., $C_i\cap C_j=\emptyset$ for any pair of cells with $i\ne j$, and $\bigcup _{1\le i\le n}C_i=V$. Each disconnected component of the graph should be partitioned into at least two cells. Within each cell $C_i$, we distinguish *border nodes* $B_i$. A node $v\in C_i$ is a border node if it has an edge to or from another cell, i.e., there is a node $w\in V, w\notin C_i$, and an edge $(v, w)\in E$ or an edge $(w, v)\in E$. For example, the temporal graph *G* in Fig. 1b of our example public transport network (Fig. 1a) can be partitioned into two cells (dashed boxes): $C_1$ with border nodes $v_5$ and $v_7$, and $C_2$ with border nodes $v_8$ and $v_9$.

The cells define the structure of the reachability index. The index will be expanded cell by cell to answer reachability queries. A good partitioning should satisfy the following properties:

1. *Well connected inside.* A cell comprises highly-linked nodes with many edges and connections inside the cell.
2. *Loosely connected outside.* The number of border nodes per cell is small.
3. *Large distance between cells.* Crossing cell borders is expensive: the number of connections between cells is small and their cost is high.

Finding a good partitioning that satisfies our requirements is not straightforward. In our scenario, the number of partitions or their sizes is not known up front, which renders many partitioning techniques inapplicable. We propose to use the Louvain method for community detection [7], which produces good partitions in our experiments. This technique efficiently finds communities in a network. It partitions the graph into communities of strongly connected nodes; nodes from different communities are loosely connected. The quality of the partitions is the so-called modularity that measures the density of links inside a community as compared to links between communities. Louvain iteratively finds good communities by increasing the modularity value. It starts with each node being in a different community and improves by moving nodes between communities. It supports a custom weight function for the links between nodes. We chose the number of connections between nodes for the weight function, i.e., how many times, according to the schedule, one can cross a direct edge between two nodes. Such a weight results in cells that are well connected inside and are loosely connected to other cells.

Exploring alternative weight functions and partitioning techniques is certainly a worthwhile effort. A possible weight refinement uses edge costs and assigns higher weights to edges with lower traversal cost. Interesting alternative graph partitionings include METIS [16] and the Merging-Algorithm [11].

### Constructing the Index Core

Given a temporal graph $G=(V,E,c)$ and a partitioning *C* of *G*, we construct the core of our reachability index. The index core is independent of POIs and never changes. The reachability index is a temporal graph $R=(V_R,E_R,c_R)$ with nodes $V_R\subset V$, edges $E_R\subseteq V_R\times V_R$, and cost function $c_R(e, t)$ on the edges $e\in E_R$. For an edge $e=(u,v)\in E_R$, $c_R$ returns the shortest-path cost from *u* to *v* at time *t*, i.e., $c_R(e, t) = sp(u,v,t)$.

*Index Nodes.* For each cell $C_{i}\in C$, we insert all its *border nodes* $B_{i}$ into the node set $V_{R}$ of the index. Thus, the nodes of the index $V_{R} = \bigcup _{1\le i\le |C|} B_{i}$. Figure 1c shows the index core of the temporal graph (Fig. 1b) with cells $C_1=\lbrace v_5, v_7\rbrace$ and $C_2=\lbrace v_8, v_9\rbrace$.

*Index Edges.* The edges of the index core are $E_R= BB \cup BC$. $BB$ is all edges between border nodes of neighboring cells. For each edge $(u,v)\in E$ between two border nodes of different cells in *C*, $u\in C_i,v\in C_j,i\ne j$, insert a new edge between the respective nodes into the index, $E_R=E_R\cup \{(u,v)\}$. *BC* is edges between pairs of border nodes within a cell. For each pair $u,v\in B_i$, insert two new edges (*u*, *v*) and (*v*, *u*) into the index, $E_R=E_R\cup \{(u,v), (v, u)\}$. For example, $BB = \lbrace \alpha _{3}, \alpha _{4}, \alpha _{7}\rbrace$ and $BC = \lbrace \alpha _{1}, \alpha _{2}, \alpha _{5}, \alpha _{6}\rbrace$ in Fig. 1c.

### Computing the Index Cost Function

The cost function $c_R$ of an edge $e=(u,v)\in E_R$ in index *R* is defined as the shortest-path cost from *u* to *v* at time *t* in graph *G*, i.e., $c_R(e,t)=sp(u,v,t)$. For computing the values of the cost function $c_R$, we execute Dijkstra’s single-source shortest-path algorithm once for every border node $b\in B_i$ and every departure time at *b*. The expansion stops when all other border nodes in the cell and all direct neighbors of *b* (i.e., nodes reachable from *b* via a $BB$ edge) are visited. Since the cells are small compared to the overall graph, typically only a small number of nodes needs to be considered for each execution of Dijkstra’s algorithm. $BC$ and $BP$ edges may connect nodes that are not reachable in the original temporal graph. If a node is not reached during one of the shortest-path computations, we assign infinite cost to the respective edges. Cost examples for the index core in Fig. 1c are: $c_R(\alpha _{3},14)=3$, $c_R(\alpha _{4},9)=3$, $c_R(\alpha _{7},8)=3$, $c_R(\alpha _{1},9)=2$, $c_R(\alpha _{2},11)=3$, $c_R(\alpha _{5},9)=2$, $c_R(\alpha _{6},8)=2$.

### Points of Interest

POIs can be inserted and deleted at any time, also after index construction. This is beneficial because POIs may change over time. A POI $v\in V$ may be any node in the original temporal graph. If *v* is a border node, no action is required because such a node is in the index core already. Otherwise, similarly to border nodes, inserting *v* into the index involves three steps. (1) We add *v* to the index nodes ($V_R=V_R\cup \{v\}$). (2) We add an edge from each border node of $v's$ cell to *v* (we call such edges BP edges). (3) The cost function based on shortest paths (like for all other edges) is computed. Deleting a POI from the index removes the POI node and all its incoming edges. For example, consider inserting two POIs, $v_{2}, v_{10}$, into the index in Fig. 1d. We add edges $BP = \lbrace \alpha _{8}, \alpha _{9}, \alpha _{10}, \alpha _{11}\rbrace$ with cost examples $c_R(\alpha _{8},9)=3$, $c_R(\alpha _{9},8)=7$, $c_R(\alpha _{10},9)=1$, and $c_R(\alpha _{11},11)=1$.

### Index Size

The index consists of border nodes and POIs. Thus, the number of index nodes is at most the number of nodes in the temporal graph. We introduce three types of edges into the index. $BB$ edges connect border nodes between different cells, and they are a subset of the temporal graph edges. $BC$ edges connect border nodes in a single cell, and their cardinality is at most quadratic in the number of border nodes. Each POI adds as many BP edges as border nodes in a cell. The numbers of $BC$ and $BP$ edges depend only on the subset of temporal graph nodes that are in a single cell. The numbers do not depend on the graph size. In sparse graphs, where many nodes have only a few edges, the reachability index may grow larger than the temporal graph: we can remove only a small number of original edges but need to insert new $BC$ and $BP$ edges.

Each edge has as many edge cost values as there are departure times from a node. The edge costs are computed for each single cell in isolation, making parallel computation possible. In particular, the edge cost of a specific border node at a specific departure time is independent of all other edge costs.

### Index Compaction

The index size, as well as the size of the temporal graph, is dominated by the size of the schedule, i.e., the number of edge connections. After computing the edge costs in the index, we observe that many different departure times have the same arrival time at the destination. It is enough to keep only one connection per arrival time, namely the one with the maximum departure time. We leverage that and compact the index by reducing the number of connections as follows. Consider an edge $e(u,v)\in E_R$ and set *S* of departure–arrival connection pairs (*d*, *a*) on that edge. We compact *S* to $S'\subseteq S$, such that $S'=\{ (d,a)\in S : \not \exists _{(d_i,a_i)\in S} a_i=a \wedge d_i>d \}$. Experiments show that this compaction technique is highly effective and reduces the index size by up to 73% (cf. Sect. 7). For example, the set of all connections on edge $\alpha _{8}$ in Fig. 1d, $\lbrace (8,12), (9,12), (11,15)\rbrace$, is compacted into $\lbrace (9,12), (11,15)\rbrace$.

## Answering Reachability Queries

The core idea of our reachability algorithm is to expand cell by cell rather than edge by edge. The $BB$ edges between border nodes of different cells allow us to expand to the neighboring cells; the $BC$ edges between border nodes of the same cell reflect the time to cross a cell; the direct $BP$ edges from border nodes to POIs allow for a quick evaluation of which POIs can be reached. In addition, we discuss a heuristic to avoid unnecessary edge expansions and processing of query nodes that are non-border nodes.

*The Reachability Algorithm.* Algorithm 1, takes as an input the reachability index $R=(V_R,E_R,c_R)$, query node *q*, start time $t_s$, and the cost budget $\varDelta t$. The expansion proceeds like in Dijkstra’s algorithm and returns the set *N* of reachable POIs in *R*. Nodes and their costs from *q* are stored in a min-heap *M* initialized to $M[q]=0$, and $M[v]=\infty$ for all other nodes *v* (line 1). The closest node *v* to *q* is popped from the min-heap (line 4), and the costs for nodes adjacent to *v* are updated if smaller (lines 9–11). To retrieve the correct edge cost, we do a binary search in the list of edge costs sorted by departure time (line 9). Each node is traversed only once. The algorithm terminates when no more nodes with cost lower than the budget are in the heap (line 5). Consider the reachability index in Fig. 1d. Here, $RQ(R,v_5,8,6)=\{v_2,v_{10}\}$ because $sp(v_{5},v_{2},t)=4$ (through $\alpha _{8}$) and $sp(v_{5}, v_{10},t)=5$ (through $\alpha _{4}$ and $\alpha _{10}$). $RQ(R,v_5,6,6)=\{v_2\}$ because $sp(v_{5},v_{2},t)=6$ (through $\alpha _{8}$) but $sp(v_{5},v_{10},t)=7$ (through $\alpha _{4}$ and $\alpha _{10}$).
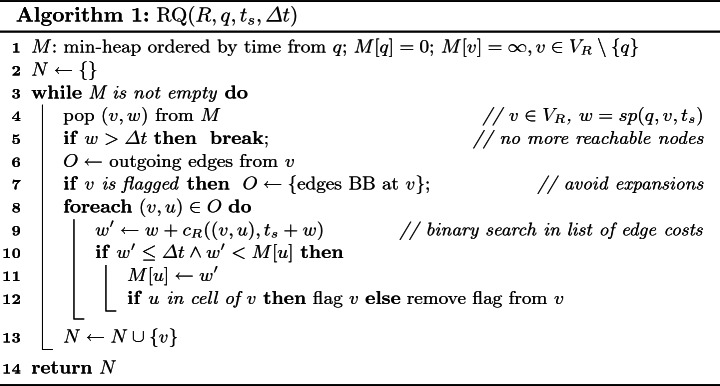


*Avoiding Unnecessary Expansions.* Regarding the edges within a cell, we observe the following. Consider Algorithm 1 processing a border node *b* of a cell $C_i$. Then, the costs of the other nodes, $v_j\in C_i$, are updated w.r.t. the cost of reaching them from *b*. When we pop a node $v_j$ in a later round, and if $v_j$ was last updated by *b*, there is no point in following the edges from $v_j$ to the other nodes in the cell. The cost of accessing the other nodes in the cell through $v_j$ cannot be smaller than the cost of accessing these nodes directly from *b* since all edge costs are shortest paths. If, however, $v_j$ was updated through an edge from a neighboring cell, the edges to the other nodes in the cell need to be followed. We exploit this observation to avoid following edges inside a cell that cannot lead to an update and thus do not affect the solution. We flag the nodes whenever their cost was updated by processing a node from within a cell, and we remove the flag, otherwise (line 12). The outgoing edges that must be expanded are selected based on the flag (line 7).

Note that the number of edges within a cell is quadratic in the number of border nodes of that cell. Thanks to the use of flags we avoid unnecessary expansions. In particular, if the cheapest way to reach all nodes in a cell is through *k* border nodes, we only expand $k(w-1)$ edges per cell, where *w* is the number of all border nodes and POIs in a cell. The value of *k* is expected to be small and will often be 1 (i.e., the shortest path from a query node *q* to all nodes in the cell crosses the border node that is closest to *q*).

*Non-border Query Nodes.* The reachability index does not contain all nodes of the original graph. If the query node *q* in cell $C_i$ is not a border node, the algorithm starts the expansion from *q* in the temporal graph. All POIs reached in cell $C_i$ are part of the result. Once a border node $b'\in B_i$ is reached, the expansion continues in the index at time $t_s+\textit{sp}(q,b',t_s)$.

*Correctness.* We show that the shortest-path costs in the index and the original temporal graph are identical. Let $u,w \in V_R$ be two index nodes and $p=\langle (v_0,v_1), (v_1,v_2), \ldots , (v_{n-1},v_n)\rangle$ be the corresponding shortest path in the temporal graph, i.e., $u=v_0, w=v_n$. If there is a direct edge between *u* and *w* in the index, the shortest-path cost is the cost of that edge: this cost is precomputed using Dijkstra’s algorithm for each departure time in the original temporal graph; since our cost function is consistent (cf. Sect. 2), the edge cost is correct [17]. Otherwise, *u* and *w* are not in the same cell (all nodes in a cell are connected with an edge). So, there must be a path along index nodes $u_1, u_2, \ldots u_k \subseteq v_1, \ldots v_{n-1}$ that are all on path *p* since cells can be exited only through border nodes. We show that the cost of the index path is indeed the shortest path. Assume a node $u_i$ exists such that $sp(v_0,v_n,t) < sp(u,u_i,t) + sp(u_i,w,t) + sp(u,u_i,t)$. On a path of length two, the costs of edges $(u, u_1)$ and $(u_1, w)$ are precomputed shortest-path costs, and they are therefore correct. The assumption, however, implies that one of the edge costs could be decreased, i.e., the assumption is incorrect. This argument can be extended edge by edge to paths of arbitrary length.

## Related Work

Shortest-path and reachability queries on road networks, i.e., graphs with constant edge cost, have been studied extensively. Unfortunately, these works cannot be applied readily to public transport networks [3]. An evaluation by Bast et al. [4] shows a large performance gap between the two types of networks. This is due to the time-dependent edge costs of public transport networks, which makes the precomputation efforts of many algorithms infeasible.

Current solutions for public transport networks either rely on Dijkstra’s algorithm [10] or require heavy precomputations. Dijkstra-based approaches include isochrone algorithms for multimodal networks [6, 12]. They expand from a query point using Dijkstra’s algorithm and compute a so-called isochrone, which is the reachable portion of the network at a given point in time. Since all edges in the isochrone must be expanded, this approach does not scale to large networks.

Many works fall into the category of labeling approaches. The earliest work, 2-hop labeling [9], is designed for weighted graphs and is based on 2-hop covers of shortest paths. Recent works strive to decrease the index size and construction time [8, 15], which are bottlenecks of 2-hop labeling and prevent application to large graphs. Time Table Labeling (TTL) [20] and Top Chain [21] adapt 2-hop labeling to public transport networks; they support shortest-path and point-to-point reachability queries. In TTL, the main idea is to precompute label sets for each node *v* containing reachable nodes from and to *v*. Top Chain creates a directed acyclic graph (DAG), where each node represents a departure time, and decomposes the DAG to create the label sets. Creating label sets in both techniques requires high precomputation costs and large index sizes. To decrease the index size, Top Chain only stores *K* label sets, called chains. The index size of Top Chain for small *K* values is smaller than that of TTL, but there is no guarantee that the query results can be found using the index.

Non-labeling techniques include Scalable Transfer Patterns [5], Connection Scan Algorithm (CSA) [19], and Contraction Hierarchy for Timetables (CHT) [13]. Transfer Patterns require an expensive profile search from each node to find the optimal paths to all other nodes. CSA organizes a schedule as two sequences of edges. The first sequence contains sorted edges based on arrival times, and the second sorts edges based on departure times. These approaches involve expensive precomputations or large index sizes, which limits their scalability.

To compute reachability queries as defined in this paper, all techniques based on point-to-point queries require the computation of shortest paths from a given query node to every POI, which does not scale to large number of POIs.

**Table 1.** Statistics of our datasets

Dataset	#Nodes	#Edges	#Conn	#Part	#B-nodes		Part. size			#POIs	
					sum	avg	avg	min	max	sum	avg
*Zurich*	2,508	5,630	555,713	45	315	7.0	55	2	157	99	2.20
*Berlin*	12,984	34,791	1,348,070	50	1,241	24.8	259	2	921	567	11.34
*Synthetic*	145,188	433,272	31,042,468	44	1,245	28.3	3,299	831	4,037	7,176	163.00

## Experiments

We experimentally evaluate our solution, *RQ*, and compare it with two competitors, a no-index solution, *NI*, and a fully-indexed solution, *SP*. We report on the index size and efficiency of the algorithms w.r.t. the number of expanded edges, which is the work that an algorithm has to do to find reachable nodes. The algorithms are implemented in Python 3 and executed on a Intel Xeon server (E5-2630 v3 2.40 GHz, 2 CPUs of 8 cores, 96 GB RAM, Debian 9.12).

*Competitors.* The no-index solution, *NI*, operates on the original temporal graph and does not build an index. The reachability is computed with a modified version of Dijkstra’s algorithm that supports our cost function (cf. Sect. 5). The fully-indexed solution, *SP*, stores all shortest paths from every node in the temporal graph to all POIs at every departure time. *SP* represents the collection of works that index the shortest paths between pairs of nodes (cf. Sect. 6).

*Datasets.* We use two real-world public transport networks represented as temporal graphs, *Zurich* and *Berlin* [1], and one synthetic graph, *Synthetic*. *Zurich* and *Berlin* are obtained in GTFS format that is further processed. For these graphs, we chose all transport modes and all connections operating on Mondays. *Synthetic* is a $6\times 6$ grid of equally-sized spider-web subgraphs. Each spider-web subgraph has one edge to every neighboring subgraph (to its left, right, top, and bottom). This graph simulates loosely connected cities that are densely connected inside. Table 1 shows the statistics. Here, #Conn is the number of all connections (departure-arrival pairs) that can be used to cross an edge. We report the details of partitioning the data graphs using the Louvain method (with maximum partition sizes): number of partitions, number of border nodes (sum and average per partition), partition sizes (avg, min, and max). We also show the number of POIs (sum and average per partition). POIs are chosen randomly as 5% of the nodes of each partition (at least one per partition).

**Table 2.** Index details

Dataset	Algorithm	#Nodes	#Edges	#Connections
*Zurich*	*RQ*	414	4,021	421,268
	*SP*	2,508	248,292	55,015,587
	*NI*	2,508	5,630	555,713
*Berlin*	*RQ*	1,808	53,543	2,533,940
	*SP*	12,984	7,361,928	764,355,690
	*NI*	12,984	34,791	1,348,070
*Synthetic*	*RQ*	8,421	212,564	18,018,811
	*SP*	145,188	1,041,869,088	222,760,750,368
	*NI*	145,188	433,272	31,042,468

*Index Size.* *RQ* and *SP* precompute certain shortest paths and build an index structure that is sufficient to answer reachability queries. If the index of *SP* is stored as a graph, its number of nodes equals #Nodes (POIs are nodes of the graph), its number of edges equals #Nodes $\times$ #POIs (shortest paths from every node to every POI are computed), and the number of connection equals #Conn $\times$ #POIs (a shortest path at every departure time to every POI is computed); #Nodes, #POIs, and #Conn are of the original temporal graph. Although *NI* does not require precomputation, the input graph has to be kept in memory. In Table 2, we compare the index sizes (*RQ*, *SP*) to the input graph size (*NI*). The values that increase the index size are the number of nodes and edges, and the number of connections. The index size of *RQ* is always smaller than that of *SP* (up to four orders of magnitude). *RQ* is also significantly smaller than the original *Zurich* and *Synthetic* graphs (*NI*). For *Berlin*, despite it having significantly fewer nodes, the numbers of edges and connections in *RQ* are larger than in the original graph. This is caused by the sparsity of *Berlin* (cf. Sect. 4.5). Finally, #Connections is the number of edge connections stored. For *RQ*, we list the absolute number of connections after the compaction. The reduction rate of compaction varies from 67% in *Synthetic* to 73% in *Zurich* and *Berlin*.

**Fig. 2.**
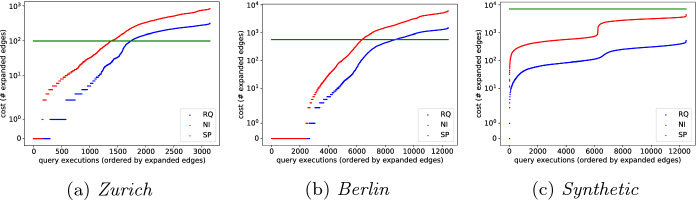
Number of expanded edges (y-axis in log scale).

*Number of Expanded Edges.* To evaluate the efficiency, we compare the number of edges that an algorithm has to process in order to find all reachable POIs (Fig. 2). One data point in the figure (scatter plot) is a single reachability query. Data points are sorted along the x-axis by the number of expanded edges. The number of expanded edges (y-axis) is displayed in log scale. We execute one reachability query starting at every border node in our index. We do so at five different start times (8:00, 12:00, 16:00, 18:00, 22:00) and for two time budgets (60 and 120 min). Thus, the number of data points is $10\times$ #Border nodes. The budgets are large enough to force *RQ* to traverse multiple edges. Since the edge costs of large cells in the *RQ* index are often above 15 min (and above 30 min in about half of the cases), budgets near these values provide little insight. Since *SP* precomputes the path to each POI, it always evaluates one edge per POI. This is a lower bound on the cost of any point-to-point index. Although the index of *SP* is orders of magnitude larger, *RQ* expands significantly fewer edges for many of the data points. We observe the largest differences for the budget of 120 min. On *Synthetic*, the number of edges expanded by *RQ* is up to three orders of magnitude lower than that of *SP*, and it is up to one order of magnitude lower than that of *NI*. *RQ* always expands fewer edges than *NI*. Values equal to zero indicate that an algorithm cannot expand due to high connection costs. We also performed similar experiment with an increased percentage of POIs (more than 5%): the difference in the number of expanded edges between *RQ* and *NI* decreases. This is to be expected since *RQ* can leverage the sparsity of POIs, while *NI* cannot.

Overall, our experiments show that dispite its small size, *RQ* substantially reduces the number of edges (by about an order of magnitude in realistic settings) and therefore speeds up reachability queries in public transport networks.

## Conclusion

The paper offers improved support for reachability queries in temporal graphs that retrieve all reachable points of interest (POIs) from a given query node at a specific start time within a given time budget. We observe that current solutions do not scale to large network (solutions based on Dijkstra’s algorithm without a pre-computed index) or to networks with many POIs (solutions based on an index for single-path queries that must be executed for each POI separately). We propose a solution based on a novel access structure, the reachability index. This index partitions the original temporal graph into cells, thus enabling us to expand the graph cell by cell rather than edge by edge. We report on experiments that suggest that our technique is both effective and efficient.
